# Supplementary material for: Magnetization jump in one dimensional J − Q2 model with anisotropic exchange
Source: Sci Rep. 2017 Dec 22;7:18104. doi: 10.1038/s41598-017-17887-w (PMC5741776; doi:10.1038/s41598-017-17887-w)
Supplement: Supplementary file 1 — Supplementary Information [file 41598_2017_17887_MOESM1_ESM.pdf]

Magnetization jump in one dimensional $J-Q_2$ model with anisotropic exchange

Bin-Bin Mao,[1] Chen Cheng,[2] Fu-Zhou Chen,[1] and Hong-Gang Luo[1,2,*]

[1]*Center of Interdisciplinary Studies & Key Laboratory for Magnetism and Magnetic Materials of the Ministry of Education, Lanzhou University, Lanzhou 730000, China*

[2]*Beijing Computational Science Research Center, Beijing 100193, China*

I. HAMILTONIAN IN THE FEW MAGNON LIMIT

Note that in this study the macroscopic magnetization jump always happens from a finite magnetization to the saturated magnetization state, which is the ferromagnetic state. Therefore, to understand the magnetization jump in the simplest way, it is natural to consider the one- and two-magnon states on a ferromagnetic background. The ferromagnetic state, which is a zero-magnon state, is denoted as $|0\rangle = |\uparrow\uparrow\uparrow\cdots\rangle$. In the Following, we examine the ground state for the system in N-magnon sectors in the few magnon limit (up to $N=2$), neglecting the contribution of the external field. For simplicity, we denote the Hamiltonian as

$$H = H_J + H_Q, \tag{S1}$$

where $H_J = -J\sum_i P_{i,i+1}$ and $H_Q = -Q\sum_i P_{i,i+1}P_{i+2,i+3}$. For the system described by the Hamiltonian in Eq. (S1), with size L and periodic boundary conditions (PBC), the energy of this zero-magnon state is

$$E(0) = -J\frac{(1-g)}{4}L - Q\frac{(1-g)^2}{16}L. \tag{S2}$$

The one-magnon excited state with momentum k can be defined as

$$|k\rangle = \frac{1}{\sqrt{L}}\sum_{l=1}^{L} e^{ikl}S_l^- |0\rangle\,. \tag{S3}$$

By acting the Hamiltonian on the state, we get

$$H_J|k\rangle = J\cos k\,|k\rangle - \frac{J(1-g)L}{4}|k\rangle - Jg\,|k\rangle \tag{S4}$$

for J term, and

$$H_Q|k\rangle = \left[\frac{Q(1-g)}{2}\cos k - \frac{Q(1-g)^2 L}{16} + \frac{Q\left(g^2-g\right)}{2}\right]|k\rangle \tag{S5}$$

for Q term. Notice that H is diagonal in $|k\rangle$ basis, we can easily obtain the energy dispersion of the system with one-magnon

$$E_k(1) = \left[J + \frac{Q}{2}(1-g)\right]\cos(k) - Jg + \frac{Q\left(g^2-g\right)}{2} - \frac{J(1-g)L}{4} - \frac{Q(1-g)^2 L}{16}. \tag{S6}$$

As in the main text, define N magnon excitation energy as $\tilde{E}(N) = E(N) - E(0)$. The one-magnon excitation energy is

$$\tilde{E}(1) = \begin{cases} -J(1+g) - \frac{Q\left(1-g^2\right)}{2} & \text{if } g < \frac{2J}{Q}+1, k=\pi \\ J(1-g) + \frac{Q(1-g)^2}{2} & \text{if } g > \frac{2J}{Q}+1, k=0. \end{cases} \tag{S7}$$

---

*Electronic address: luohg@lzu.edu.cn

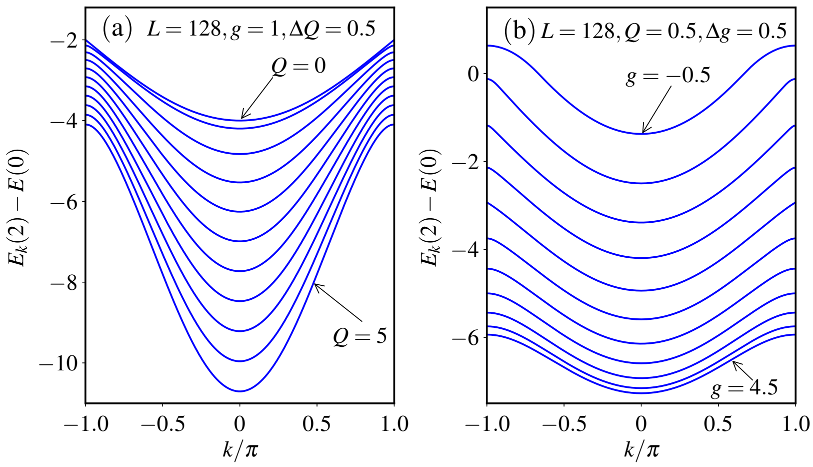

Fig. S1: (Color online) The dispersion of the two-magnon exited states of (a) $g = 1$ for different Q, and (b) $Q = 0.5$ for different g. Here system size $L = 128$.

Here $\tilde{E}(1)$ can also be considered as the energy of a free magnon.

For two-magnon state, we choose the basis with a total momentum k and a relative distance d defined as

$$|d,k\rangle = \frac{1}{\sqrt{L}}\sum_l e^{ik(2l+d)/2} S_l^- S_{l+d}^- |0\rangle \,. \tag{S8}$$

Acting the Hamiltonian on the basis set, for the J term, we obtain:

$$H_J|1,k\rangle = \left(\frac{g-1}{4}JL - Jg\right)|1,k\rangle + J\cos\frac{k}{2}|2,k\rangle\,, \tag{S9}$$

$$H_J|d>1,k\rangle = J\cos\frac{k}{2}\left(|d+1,k\rangle + |d-1,k\rangle\right) + \left(\frac{g-1}{4}JL - 2Jg\right)|d,k\rangle\,. \tag{S10}$$

For the Q term, there are

$$H_Q|1,k\rangle = \left[-\frac{(1-g)^2 QL}{16} + \frac{(g^2-2g)Q}{4}\right]|1,k\rangle + \frac{Q}{2}\cos\frac{k}{2}|2,k\rangle - \frac{Q}{4}|3,k\rangle\,, \tag{S11}$$

$$H_Q|2,k\rangle = \frac{Q}{2}\cos\frac{k}{2}\left(|3,k\rangle + |1,k\rangle\right) + \left[-\frac{(1-g)^2 QL}{16} + \frac{(g^2-2g-\cos k)Q}{2}\right]|2,k\rangle\,, \tag{S12}$$

$$H_Q|3,k\rangle = -\frac{Q}{4}|1,k\rangle + \frac{Q}{2}\cos\frac{k}{2}|2,k\rangle + \left[-\frac{(1-g)^2 QL}{16} + Q\left(\frac{3}{4}g^2 - g\right)\right]|3,k\rangle + \frac{Q(1-g)}{2}\cos\frac{k}{2}|4,k\rangle\,, \tag{S13}$$

$$H_Q|d>3,k\rangle = \frac{Q(1-g)}{2}\cos\frac{k}{2}\left(|d+1,k\rangle + |d-1,k\rangle\right) + \left[-\frac{(1-g)^2 QL}{16} + Q\left(g^2-g\right)\right]|d,k\rangle\,. \tag{S14}$$

Then the ground state energy $E_2(k)$ of the two-magnon state with momentum k can be obtained by numerically diagonalizing the $(L-1)\times(L-1)$ Hamiltonian matrix in the basis set $|d,k\rangle$. In Fig. S1 we show the dispersion $E_k(2) - E(0)$ of the two-magnon exited states for several different parameters. As one can see, for these examples the two-magnon ground state always has $k = 0$. Actually, we have carefully checked the dispersion for all the parameters

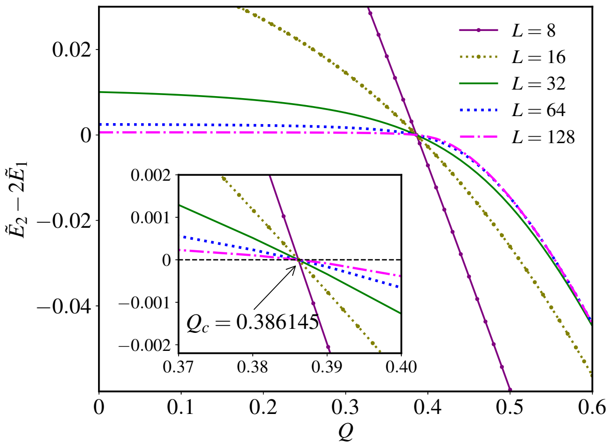

Fig. S2: (Color online) $\tilde{E}(2)-2\tilde{E}(1)$ as a function of Q for $g=0.5$ and different L. The black dashed line in inset is for $\tilde{E}(2)-2\tilde{E}(1)=0$.

we concern in this work, and the minimum $E_k(2)-E(0)$ for each point in the parameter space always has zero momentum. Therefore, the Hamiltonian matrix $\tilde{H}(2)=H_J+H_Q-E(0)$ in the two-magnon basis can be simplified as

$$\tilde{H}(2)=\begin{pmatrix} Q\frac{g^2-2g}{4}-Jg & \frac{Q}{2}+J & -\frac{Q}{4} & & \\ \frac{Q}{2}+J & Q\frac{g^2-2g-1}{2}-2Jg & \frac{Q}{2}+J & & \\ -\frac{Q}{4} & \frac{Q}{2}+J & Q\frac{3g^2-4g}{4}-2Jg & \frac{Q(1-g)}{2}+J & \\ & & \frac{Q(1-g)}{2}+J & Q\left(g^2-g\right)-2Jg & \\ & & & & \ddots \end{pmatrix}. \tag{S15}$$

In order to see the finite size effect in the few magnon limit, we plot $\tilde{E}(2)-2\tilde{E}(1)$ for $g=0.5$ as a function of Q for different system sizes, as shown in Fig. S2. All these curves have a precise cross at $\tilde{E}(2)-2\tilde{E}(1)=0$ and a critical $Q_c(g=0.5)=0.386145$ even for $L=8$, which is the minimum system size to include all the information of the effective Hamiltonian described by Eq. (S15). Therefore, the finite size effect in the few magnon limit is negligible.

II. THE ASYMPTOTIC BEHAVIOR

From Fig.3(c) in the main text we can see that the phase boundary between the N-MJ and PF-MJ phases can be exactly determined by comparing the energy of one- and two-magnon excitations. The phase boundary obtained in the few magnon limit perfectly agrees with the numerical results by DMRG.

We also notice the asymptotic behavior of this curve when the pair coupling Q is extremely large. In the limit of $Q\to\infty$, the one-magnon excitation energy is

$$\tilde{E}(1)/Q=\begin{cases} -\frac{1-g^2}{2} & \text{if } g<1, \\ \frac{(1-g)^2}{2} & \text{if } g>1. \end{cases} \tag{S16}$$

here we have ignored the infinite small terms proportional to $1/Q$. Similarly, ignoring the $O(1/Q)$ terms, the two-magnon excitation is described by the matrix

$$\tilde{H}(2)/Q=\begin{pmatrix} \frac{g^2-2g}{4} & \frac{1}{2} & -\frac{1}{4} & & \\ \frac{1}{2} & \frac{g^2-2g-1}{2} & \frac{1}{2} & & \\ -\frac{1}{4} & \frac{1}{2} & \frac{3g^2-4g}{4} & \frac{1-g}{2} & \\ & & \frac{1-g}{2} & g^2-g & \\ & & & & \ddots \end{pmatrix}. \tag{S17}$$

We can numerically obtain the critical anisotropy g_c satisfying $\tilde{E}(2)-\tilde{E}(1)=0$. For the ground state wave function ($|G\rangle_2=\sum_{d=1}^{L-1}\alpha_d\,|d\rangle$) at the critical point, we find that i)α_d^2 is a constant number, ii) $\alpha_{d+1}=-\alpha_d$, for d and $d+1$ in range $[3,L-3]$. Thus, the critical wave function can be assumed as

$$|G\rangle_2=\frac{1}{\eta}\left[a\,|1\rangle+b\,|2\rangle+c\,|3\rangle+\sum_{d=4}^{L-4}(-1)^d\,|d\rangle+c\,|L-3\rangle+b\,|L-2\rangle+a\,|L-1\rangle\right], \tag{S18}$$

where $|d\rangle\equiv|d,k=0\rangle$, η is the normalization coefficient.

Applying the Hamiltonian in Eq. (S17) to the wavefunction $|G\rangle_2$,we can get a set of equations. By solving them, the critical g in the $Q\to\infty$ limit can be obtained as $g_c(Q\to\infty)=\left(-4+\sqrt{7}\right)/3$. For any anisotropy g below this value, the magnetization curve of the system is always smooth and continuous.

III. THE EFFECTIVE HAMILTONIAN IN LARGE ANISOTROPY LIMIT

Divided by g^2 on both sides, the Hamiltonian in this study can be written as

$$\frac{H}{g^2}=-Q\sum_i S_i^zS_{i+1}^zS_{i+2}^zS_{i+3}^z+O\left(\frac{1}{g}\right)+O\left(\frac{1}{g^2}\right)-h'\sum_i S_i^z, \tag{S19}$$

where

$$h'=\frac{h}{g^2}, \tag{S20}$$

$$\begin{aligned}O\left(\frac{1}{g}\right)=&\frac{1}{g}\Bigg\{J\sum_i S_i^zS_{i+1}^z\\&+Q\sum_i S_i^zS_{i+1}^z\left[\frac{1}{4}-\frac{1}{2}\left(S_{i+2}^+S_{i+3}^-+S_{i+2}^-S_{i+3}^+\right)\right]\\&+Q\sum_i\left[\frac{1}{4}-\frac{1}{2}\left(S_i^+S_{i+1}^-+S_i^-S_{i+1}^+\right)\right]S_{i+2}^zS_{i+3}^z\Bigg\},\end{aligned} \tag{S21}$$

$$\begin{aligned}O\left(\frac{1}{g^2}\right)=&\frac{1}{g^2}\Bigg\{-J\sum_i\left[\frac{1}{4}-\frac{1}{2}\left(S_i^+S_{i+1}^-+S_i^-S_{i+1}^+\right)\right]\\&-Q\sum_i\left[\frac{1}{4}-\frac{1}{2}\left(S_i^+S_{i+1}^-+S_i^-S_{i+1}^+\right)\right]\left[\frac{1}{4}-\frac{1}{2}\left(S_{i+2}^+S_{i+3}^-+S_{i+2}^-S_{i+3}^+\right)\right]\Bigg\}.\end{aligned} \tag{S22}$$

In the limit of $g\to\infty$, Q is finite, the $O(1/g)$ and $O(1/g^2)$ terms can be neglected . Thus the effective Hamiltonian in the large anisotropy limit is

$$\mathscr{H}_{g\to\infty}=-Q\sum_i S_i^zS_{i+1}^zS_{i+2}^zS_{i+3}^z-h'\sum_i S_i^z. \tag{S23}$$

IV. THE CORRELATION FUNCTION

In this section we discuss the different behaviors of the spin-spin correlation function in the experienced sectors and the *jumped-over* sectors. Fig. S3 displays the correlation function $C_S(r)$ with different parameters as examples. In the N-MJ phase ($g=-0.5$), independent of the magnon number N, the spin-spin correlation $C_S(r)$ is negative for all the distance $r>0$, and rapidly decays to 0 as r increases. In this case, a spin has anti-ferromagnetic correlation with

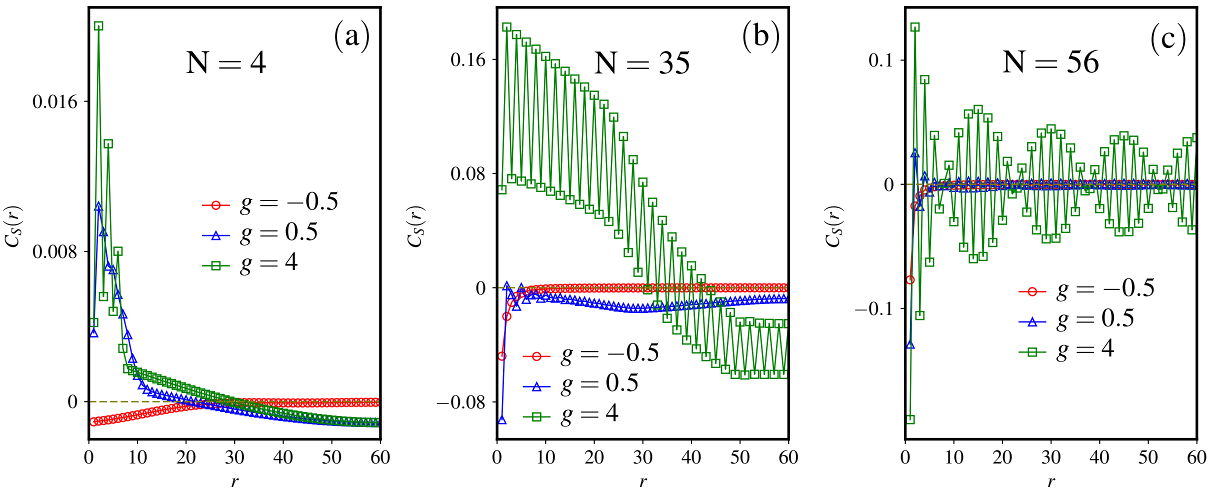

Fig. S3: Spin-spin correlation function $C_S(r)$ for different g and N. Here $Q = 1.5$ and $L = 120$.

its environment, and is screened due to the strong quantum fluctuation. The states in the experienced sector have no long-range order (LRO).

In the PF-MJ phase ($g = 0.5$), the magnetization curve is continuous at some magnetization density, and then has a sharp jump. The blue triangle-line shown in Fig. S3(a) for $N = 4$ is the correlation function of an *jumped-over* state. In this state, $C_S(r)$ is positive when the distance r is small but negative when the spins are far apart from each other, which is the typical behavior of the system having two ferromagnetic domains. We also notice $C_S(r)$ has a finite value even when $r = L/2$, which is the largest distance possible for the finite system with system size L. In fact, $C_S(r)$ seems to converge when r is large enough. This indicates the anti-ferromagnetic (AFM) long-range order of the *jumped-over* states. The AFM-LRO still can be observed when $N = 35$, as this state is also jumped over in the magnetization process, as the blue triangle-line shown in Fig. S3(b). Further increasing the magnon number N, the correlation function $C_S(r)$ of the 56-magnon state decays rapidly to zero, similar to the situation in the N-MJ phase. The AFM-LRO disappears, and the magnetization curve in this region is continuous.

The correlation functions are more complicated in the NF-MJ phase ($g = 4.0$). Since the anisotropy g is sufficient large, the diagonal term of the Hamiltonian dominates and the quantum effect has been partially depressed. We can observe the strong fluctuation of $C_S(r)$ at very large distance, especially when the magnon number N is large. Nevertheless, when N is small ($N = 4$ and 35), besides the fluctuations, the long-range nature of domain wall is still true at large distance. For a large $N = 56$, which is near the zero magnetization, the correlation function shown in Fig. S3(c) exhibits a classical Néel order.
